# Programming plant resilience with CRISPR/dCas9 epigenome editing: from proof of concept to crop deployment

**DOI:** 10.3389/fgeed.2026.1927877

**Published:** 2026-10-21

**Authors:** Jacob Wu

**Affiliations:** Hopkinton High School, Hopkinton, MA, United States

**Keywords:** chromatin remodeling, CRISPR/dCas9, DNA methylation, epigenetic stress memory, epigenome editing, histone modifications, stress priming, transgenerational inheritance

## Abstract

Accelerating climate change is driving unprecedented increases in the frequency and severity of abiotic stresses, threatening global food security and demanding new strategies for crop resilience. Plants have evolved a sophisticated molecular response to recurrent stress through epigenetic priming, where prior exposure induces chromatin-level alterations encoded through DNA methylation, histone modifications, and small RNA pathways. These imprints can persist somatically across cell divisions and, in some cases, be transmitted to subsequent generations. CRISPR/dCas9-based epigenome editing provides the means to install, modify, or erase these same marks at defined loci without altering the underlying sequence. This mini review provides an evidence-based survey of plant dCas9 epigenome editing, distinguishing what each effector class has actually been demonstrated to achieve *in planta* from what has been shown only in mammalian systems or remains inferred. I map the translational barriers between *Arabidopsis* proof-of-concept and crop deployment, survey what has been achieved in rice, maize, and tomato, organize the toolkit around the distinct memory logics (type I persistent versus type II poised) it must serve, and identify the priorities that would advance the field from demonstrated single-locus edits toward heritable, deployable modifications. By separating demonstrated capability from extrapolation, this synthesis provides a realistic basis for applying epigenome editing to sustainable agriculture and climate-resilient crop production.

## 1 Introduction

As sessile organisms, plants must endure environmental perturbations. As climate change accelerates the frequency, intensity, and co-occurrence of abiotic stresses like drought, heat, and salinity, the agronomic cost of inadequate stress responses grows. Anthropogenic climate change has reduced global agricultural total factor productivity by approximately 21% since 1961, erasing the equivalent of 7 years of productivity growth, with reductions of 26%–34% in already-warm regions of Africa, Latin America, and the Caribbean (Ortiz-Bobea et al., 2021). Conventional breeding cycles are slow relative to this rate of change.

Yet plants do not withstand these disturbances passively. Exposure to a sublethal stress can prime the organism with molecular traces that enable faster, stronger, or more targeted responses upon stress recurrence. This phenomenon is documented across diverse stressors and species, with its molecular basis residing in epigenetic changes such as DNA methylation, histone modifications, and small RNA populations that contribute to chromatin alterations underlying stress memory (Lämke and Bäurle, 2017; He and Li, 2018).

Most existing research in this field has been descriptive, cataloguing the marks associated with stress memory in model species such as *Arabidopsis thaliana,* with comparatively little work in staple food crops. The translational dimension is underexplored. Can we deliberately engineer stress memory to produce crops that are epigenetically pre-adapted to future stresses? CRISPR/dCas9-based epigenome editing—programmable, sequence-specific deposition or removal of chromatin marks—makes this question tractable, but only if the technology is matched to the molecular logic of plant memory, which current tools do not yet fully support. Recent reviews have covered epigenetic gene regulation and its crop applications (Zhang and Zhu, 2025), CRISPR-Cas crop engineering more broadly (Tuncel et al., 2025), plant epigenome editing mechanisms (Menelih et al., 2025), and natural epigenetic variation in breeding (Springer and Schmitz, 2017), but none has assessed the dCas9 toolkit against the specific requirements of plant stress memory.

This mini review addresses that gap, examining what each effector class has demonstrated *in planta*, where the evidence base still rests on mammalian systems, and what translational barriers separate existing single-locus proof-of-concept work from crop deployment. The article is organized around the distinction between type I and type II stress memory logic, which imposes different requirements on effector design, reviews separately what has been achieved in crop species, and closes with an assessment of the priorities most likely to advance the field.

## 2 Background

Stress priming occurs when mild exposure modifies a plant’s subsequent response to stress, with the primed plant responding faster, more strongly, or with greater sensitivity than an unexposed plant (Conrath et al., 2015). Stress memory is the retention of primed states, operating somatically (within a generation), intergenerationally, or transgenerationally. These differ mechanistically as intergenerational effects may reflect maternal provisioning rather than heritable chromatin change while transgenerational inheritance requires marks to survive meiotic resetting. This section briefly surveys the chromatin marks underlying these phenomena, framed explicitly by the class of dCas9 effector that would be required to recapitulate each.

### 2.1 DNA methylation

In plants, cytosine methylation occurs in CG, CHG, and CHH contexts (where H represents any non-G nucleotide) (Henderson and Jacobsen, 2007). *De novo* methylation is established primarily through the RNA-directed DNA methylation (RdDM) pathway, in which 24-nucleotide small interfering RNAs guide the methyltransferase *DRM2* to target loci (Law and Jacobsen, 2010; Zilberman et al., 2003). MET1 and CMT3 maintain CG and CHG methylation, enabling mitotic inheritance, while ROS1 and DME mediate active demethylation in vegetative and reproductive tissues respectively (Gong et al., 2002; Penterman et al., 2007). CG methylation can show relative stability through meiosis, with some marks surviving the germline bottleneck and providing a potential mechanism for transgenerational inheritance (Becker et al., 2011; Schmitz et al., 2011). Durable methylation-based memory therefore requires effectors that engage native RdDM or maintenance pathways rather than depositing a one-shot mark—a constraint absent from mammalian systems, where DNMT3A is the principal *de novo* enzyme.

### 2.2 Histone modifications

Two histone modification classes, H3K4me2/me3 (active marks) and H3K27me3 (repressive marks deposited by Polycomb Repressive Complexes), have emerged as central to stress memory.

(Bäurle, 2018; Turck et al., 2007; Yang et al., 2024). Stress memory genes fall into two functionally distinct types (Bäurle, 2018; Maric, 2025). These are a functional framework as individual loci may not map cleanly onto one type. Type I memory genes show persistent, altered expression throughout recovery, remaining on or off even after stress subsides. By contrast, type II memory genes return to baseline after the first stress but exhibit super-induction upon re-encounter. Di et al. (2024) used genome-wide H3K4me3 ChIP-seq in cucumber to demonstrate that this poised state corresponds to persistence of active H3K4me3 marks at type II memory gene promoters during recovery. Type I genes may instead maintain silencing through repressive H3K27me3 marks, as observed for *FLC* during cold memory (Bastow et al., 2004). However, this association is context- and locus-dependent. In heat stress memory, *HSFA2* and the histone variant H2A.Z are the key determinants (Charng et al., 2006; Coleman-Derr and Zilberman, 2012; Kumar and Wigge, 2010). Notably, the cucumber work of Di et al. (2024) and the rice SNAC1–OsERF103–OsSDG705 drought module (Yang et al., 2024) establish that this framework is not confined to *Arabidopsis*. The distinct logic of type I and type II memory has direct implications for effector choice: durable repression versus poised activation cannot be engineered with the same fusion protein, a point developed in Section 3.2.

### 2.3 Small RNAs and non-coding RNAs

Small RNAs, particularly 24-nt siRNAs and miRNAs, contribute to systemic signaling and intergenerational information transfer (Molnar et al., 2010; Junaid et al., 2024; Lewsey et al., 2016). They direct chromatin modifications at target loci via the RdDM pathway (Hamilton and Baulcombe, 1999) and can load into gametes, communicating parental stress histories to progeny. Long non-coding RNAs add further complexity; SVALKA regulates CBF1 during cold stress (Kindgren et al., 2018; Gómez-Martínez et al., 2024). Because RdDM is central, any plant methylation editor must either supply siRNA-guided machinery or hijack endogenous RdDM at the target locus, a constraint that distinguishes plant from mammalian design.

## 3 Engineering stress memory with dCas9

### 3.1 The logic of memory versus forgetting

Epigenetic resetting may represent an adaptive strategy rather than memory failure. Maintaining a primed state imposes metabolic costs, and where stress is unlikely to recur those costs outweigh the benefit (Crisp et al., 2016; van Hulten et al., 2006). Heritable memory should therefore evolve only where stress is predictable and recurrent (Bossdorf et al., 2007; Herman and Sultan, 2011; Latzel et al., 2013). Neither selection nor domestication has optimized it for modern agriculture. For example, crop–wild epigenomic comparisons show domestication-associated change at stress-responsive loci (Xu et al., 2020; Cao and Chen, 2024). Figure 1 contrasts the two routes to stress-ready crops: natural priming and dCas9-based engineering. Engineering is preferable to selection from standing variation because breeders cannot wait for beneficial epialleles to accrue, and because locus-specific deposition avoids the genome-wide side effects of chemical priming or repeated stress exposure.

**FIGURE 1:**
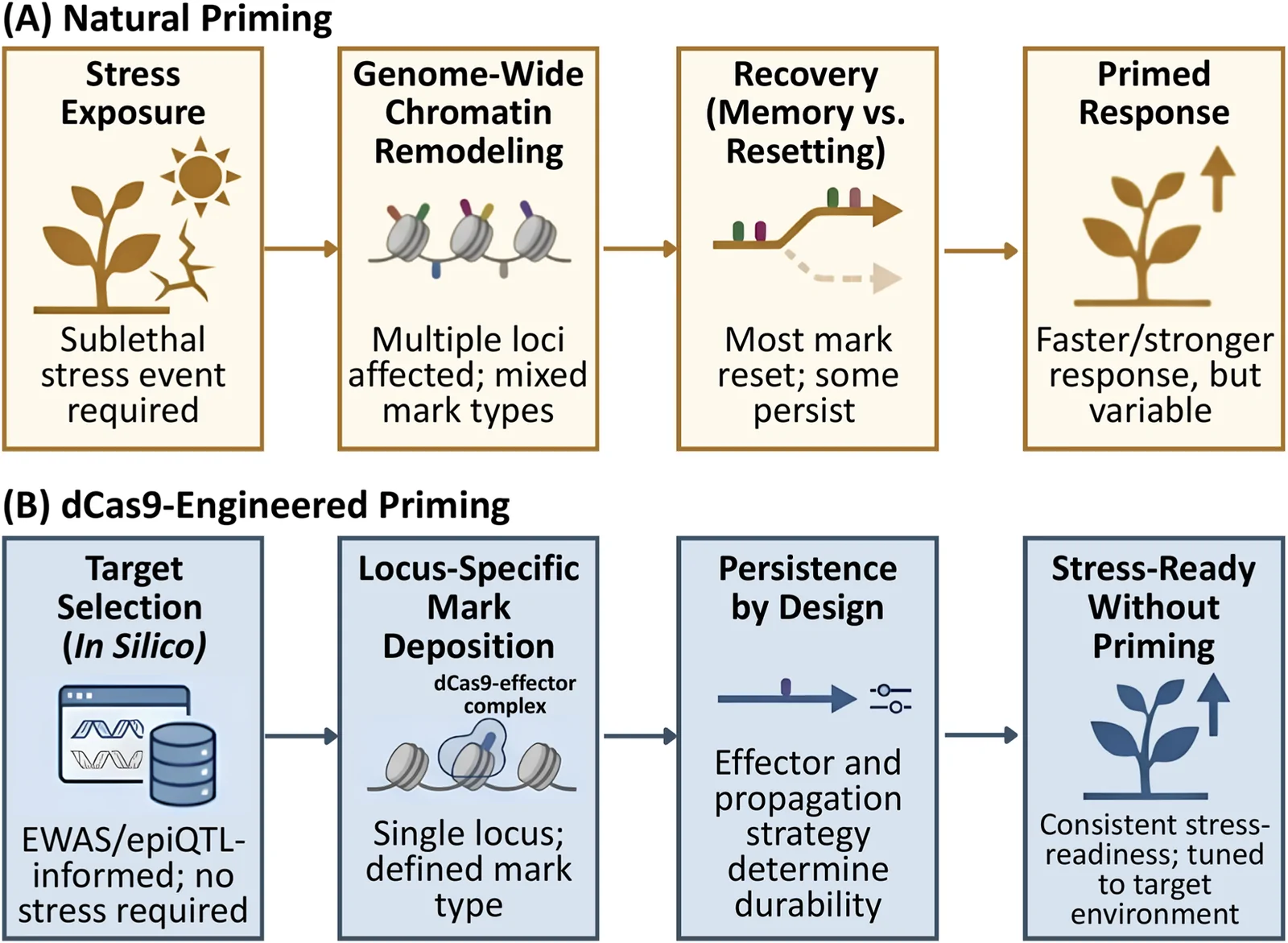
**(A)**Natural priming requires stress exposure, produces genome-wide chromatin changes of variable persistence, and yields genotype-dependent memory outcomes. **(B)** dCas9-based engineering bypasses stress exposure, deposits defined marks at single loci, and enables designed persistence through effector and propagation choices, allowing memory depth to be programmed to match target growing conditions.

### 3.2 The dCas9 epigenome editing toolkit

Catalytically inactive Cas9 (dCas9) fused to chromatin-modifying effector domains provides programmable, locus-specific control over chromatin state without altering DNA sequence (Qi et al., 2013; Villiger et al., 2024). The effector menu maps directly onto the memory marks described in Section 2, but the suitability of each fusion for engineering durable stress memory varies. Table 1 summarizes the published evidence for each effector class across Type I memory fit, Type II memory fit, and durability without continuous editor expression. Cells and the text below use the same three evidence tiers, so that plant results are not conflated with mammalian extrapolation.

**TABLE 1:** Evidence base for dCas9 effector class fit to plant stress memory engineering.

Effector class	Example fusions	Type I memory (persistent change)	Type II memory (poised state)	Durability without continuous expression	Plant-specific constraint
HAT fusions	dCas9-p300, dCas9-*AtHAC1*	Not demonstrated	Demonstrated *in planta*	Labile	Acetylation labile; opposed by endogenous HDAC activity
Histone methyltransferase fusions	SunTag-SET, dCas9-PRC2 recruiters	Not tested in plants	Demonstrated *in planta*	Not tested in plants	SET enzymes act in multiprotein complexes; simple fusion inefficient
DNA methyltransferase fusions	dCas9-*DNMT3A*/3L, SunTag-*DRM2*	Demonstrated *in planta*	Category mismatch	Mammalian only	Plant methylation is RdDM-mediated; mammalian *DNMT3A* architectures need plant adaptation
DNA demethylase fusions	dCas9-*ROS1*, dCas9-TET1	Category mismatch	Partial	Reverts (*in planta*)	RdDM re-installs mark unless concurrently suppressed
Transcriptional repressors	dCas9-KRAB, dCas9-SRDX	Demonstrated *in planta*	Category mismatch	Non-heritable alone	*KRAB-ZFP* lacks plant functional homolog; SRDX silencing not self-maintaining

Each row represents a class of dCas9 epigenome editing tool assessed across four dimensions: demonstrated suitability for type I memory (persistent expression change) and type II memory (poised, super-inducible state); durability of the installed mark without continuous editor expression; and the plant-specific constraint that distinguishes each effector from its mammalian equivalent. Cell entries report evidence status rather than performance rating. Columns 3 and 4 assess whether each effector can install the chromatin state required for the respective memory type. Column 5 assesses whether installed marks persist after editor removal, a property orthogonal to memory type suitability but essential for agronomic deployment. Column 6 identifies the molecular or biological constraint that prevents direct translation of mammalian architectures to plant systems. Full evidence and citations for each cell are provided in Section 3.2. Evidence tiers: “demonstrated *in planta*,” published in a plant system; “mammalian only,” shown in mammalian cells but not plants; “partial,” shown in plants but incompletely or at a subset of targets; “not demonstrated,” evidence does not support the property; “not tested in plants,” no published assessment. Durability terms: “labile,” the mark is intrinsically unstable; “reverts (in planta),” the mark is installed but reversed by endogenous activity; “non-heritable alone,” the modification does not propagate without continuous editor expression. “Category mismatch” indicates the effector’s mode of action is incompatible with the requirements of that memory type. For example, a transcriptional repressor cannot install the poised state that defines type II memory, so the cell is not applicable rather than merely untested.

Histone acetyltransferase (HAT) fusions. dCas9-p300 (mammalian) and dCas9-*AtHAC1* (plant) deposit H3K27ac and related activating marks at target promoters and enhancers, driving transcriptional activation (Hilton et al., 2015; Roca Paixão et al., 2019). They suit inducing a primed, transcriptionally competent state at type II promoters, but acetylation is labile, with half-lives of roughly 1–2 h at most sites (Zheng et al., 2013), requiring continuous effector expression. They therefore induce transient stress responsiveness rather than heritable memory.

Histone methyltransferase fusions. Direct deposition of H3K4me3 (type II) and H3K27me3 (type I) is attractive but technically difficult in plants. SET-domain enzymes function within multiprotein complexes whose recruitment via simple dCas9 fusion is inefficient; CRISPRa systems such as SunTag amplify recruitment but often act through indirect activation rather than direct mark deposition (Papikian et al., 2019). The García-Murillo et al. (2023) demonstration of CRISPRa-mediated H3K4me3 enrichment at *SlPR-1* in tomato indicates that effective deposition is achievable but remains genotype- and target-dependent.

DNA methyltransferase fusions. In mammalian cells, dCas9-DNMT3A and dCas9-DNMT3A/3L fusions install heritable CG methylation that propagates through cell division (Vojta et al., 2016; Stepper et al., 2017). The CRISPRoff platform couples KRAB-mediated nucleation to DNMT3A/3L methylation, giving transcriptional memory that persists through hundreds of cell divisions (Nuñez et al., 2021). Direct translation to plants is constrained because plant *de novo* methylation is RdDM-dependent rather than DNMT3A-driven, plant CHG and CHH maintenance pathways are distinct from mammalian systems, and KRAB-ZFP repressors lack functional homologs in plants. Plant-adapted approaches instead use dCas9-SunTag fused to RdDM machinery components, achieving targeted methylation with variable transgenerational inheritance (Papikian et al., 2019; Ghoshal et al., 2021).

DNA demethylase fusions. dCas9-*ROS1* fusions can reactivate silenced loci through targeted 5-methylcytosine excision (Devesa-Guerra et al., 2020), and the human TET1 catalytic domain has been deployed in *Arabidopsis* to achieve targeted demethylation (Gallego-Bartolomé et al., 2018). They erase inhibitory methylation at stress-response loci, but ongoing maintenance methylation reverses the effect unless RdDM is also suppressed.

Transcriptional repressors. KRAB and SRDX repressor domains fused to dCas9 silence target loci (Thakore et al., 2015; Tang et al., 2017). In plants, dCas9-SRDX silences robustly but may not install the stable H3K27me3 of natural type I memory, leaving silencing dependent on continuous editor presence.

Matching effector to memory logic. The two memory types require different tool classes. Type II needs durable H3K4me3 without sustained activation, which current HAT and CRISPRa fusions achieve only partially; type I needs stable methylation or H3K27me3, for which dCas9-DNMT-like and PRC2-recruiting fusions are the closest analogs but plant-specific RdDM-recruiting variants remain in development.

### 3.3 dCas9 in crop improvement

Transcriptional control platforms have translated to crops more readily than mark-depositing effectors. CRISPR-Act2.0 improved on dCas9-VP64 through a modified gRNA2.0 scaffold and was validated in Arabidopsis and rice (Lowder et al., 2018). CRISPR-Act3.0 subsequently achieved four-to six-fold stronger activation and simultaneous induction of up to seven genes in rice, tomato, and Arabidopsis, including the β-carotene and proanthocyanidin pathways in rice (Pan et al., 2021). A dedicated dCas9-VP64 and dCas9-SRDX vector set for maize allows guide RNAs to be screened in protoplasts before committing to transformation, a practical response to the recalcitrance of that species (Gentzel et al., 2020).

Chromatin-level work in crops is scarcer but no longer absent. García-Murillo et al. (2023) enriched H3K4me3 at SlPR-1 in tomato, and Tang et al. (2022) reported that targeted demethylation produced heritable epialleles in rice. Two gaps follow. First, to our knowledge, wheat, soybean, and cassava have no published dCas9 epigenome editing studies, so what is often described as a plant toolkit is, in practice, a rice, tomato, and maize toolkit. Second, reported phenotypes are almost entirely transcript abundance or survival under controlled stress; traits that determine agronomic value including photosynthetic performance, water-use efficiency, nutrient uptake, root architecture, reproductive development, and yield stability under field conditions remain essentially unmeasured (Menelih et al., 2025). Until stress phenotyping in this field extends to these physiological endpoints, claims of improved resilience rest on proxies.

### 3.4 Proof-of-concept studies and their limits

Existing studies demonstrate that targeted chromatin modification can alter stress responses. Roca Paixão et al. (2019) improved drought tolerance in *Arabidopsis* using dCas9-AtHAC1 at the *AREB1* promoter, activating downstream genes including *RD29A*. Sen et al. (2025) proposed dCas9 epigenome editing for heritable herbicide resistance without sequence alteration. The narrowness of the demonstrated locus set is itself informative. Successful targets—*FWA, AREB1, SlPR-1*—share accessible promoters, well-characterized regulation, and strong single-gene phenotypes, whereas most stress memory traits are polygenic and depend on loci whose chromatin context is unmapped in crops. Locus choice has so far been driven by tractability rather than agronomic priority. Three limitations recur. Transgenerational stability is rarely tested beyond seed set; fitness costs are assessed in growth chambers rather than the field, leaving yield under non-stress conditions unaddressed (van Hulten et al., 2006; Walters and Heil, 2007); and dependence on continuous transgene expression undercuts the goal of installing a mark and removing the apparatus, a step required for classification outside transgenic regulation in product-based regimes.

### 3.5 Design requirements emerging from the evidence base

Three design requirements follow from the evidence above. The installed mark must persist once the editing transgene is removed—shown for methylation-based systems in mammalian cells (Nuñez et al., 2021; Cappelluti et al., 2024), only partially in plants (Papikian et al., 2019). Effectors must engage endogenous RdDM biology rather than bypass it, since the mammalian DNMT3A architectures have no direct plant equivalent (Onodera et al., 2005; Ausin et al., 2009). And reversibility through paired activator systems, achieved by CRISPRoff/CRISPRon in mammalian cells, remains unexplored in plants. Specific plant-adapted fusion architectures meeting all three remain untested.

## 4 Limitations and path forward

Substantial barriers remain. First, the translation gap between model species and crops: most mechanistic data originate from *Arabidopsis*, with crop genomes posing challenges of off-target effects, delivery, and stability due to higher repetitive content (Mirouze and Vitte, 2014). Delivery of dCas9 constructs and subsequent regeneration remain species- and genotype-dependent; agrobacterium-mediated transformation and biolistic bombardment are inefficient for recalcitrant monocots, while nanoparticle delivery (Demirer et al., 2019; Cunningham et al., 2018) and ribonucleoprotein systems (Zhang et al., 2021) require further development (Prasad and Shetty, 2026). Construct size compounds this. Effector fusions, especially multiplexed SunTag architectures, produce large T-DNA cargos, and appending activation or repression domains to dCas9 measurably reduces protein accumulation *in planta* while lowering transformation efficiency (Gentzel et al., 2020). *Agrobacterium*-mediated transformation works across many crops, but efficient delivery and stable expression of these oversized constructs remain the practical bottleneck, confining recalcitrant genotypes to a few amenable lines or to transient protoplast assays.

Limited heritability is the binding constraint on agronomic use, since an epiallele that must be re-installed each generation offers no advantage over a transgene. Papikian et al. (2019) reported targeted methylation with inconsistent transmission, and the heritable rice epialleles of Tang et al. (2022) remain an exception; CHH methylation in particular decays without continued RdDM input, and no engineered mark has yet been tracked beyond F2 in a field-grown crop.

Second, stress specificity is inadequately understood. Field conditions involve simultaneous stresses, and ABA–SA–JA antagonism complicates memory engineering (Koornneef and Pieterse, 2008; Pieterse et al., 2012). ABA-mediated drought memory could inadvertently suppress SA-mediated pathogen defenses. Sustained priming imposes growth-defense trade-offs invisible in single-stress trials (Karalija et al., 2025). Third, installed marks face extensive reprogramming during flowering and seed set (Calarco et al., 2012; Iwasaki and Paszkowski, 2014); identifying meiotically stable marks is a prerequisite for transgenerational applications.

Three engineering milestones would constitute meaningful progress: (i) a plant-specific fusion that installs H3K27me3 at a type I locus and is inherited through F2 without continued editor expression; (ii) a CRISPRa system that deposits H3K4me3 at a type II memory promoter in a crop species (rice or tomato) and demonstrates super-induction upon stress re-exposure with no yield penalty under non-stress field conditions; and (iii) an RdDM-recruiting effector that installs CHH methylation at a stress-responsive promoter and propagates it through clonal generations in a crop such as potato or strawberry. Beyond tool development, EWAS and epiQTL mapping (Schmitz et al., 2013; Quadrana and Colot, 2016) and single-cell epigenomic profiling (Luo et al., 2020) will identify the highest-priority loci and resolve the germline bottleneck, and several adjacent technologies should accelerate this. Long-read sequencing calls methylation on native DNA and so resolves the repetitive regions where short-read bisulfite data fail in large crop genomes, pan-genomic references capture epigenomic variation a single reference misses, machine-learning guide design can prioritize target sites, and epigenomic selection would let validated epialleles enter breeding pipelines alongside genomic selection.

Translation also requires navigating regulation. The United States and United Kingdom, with product-based frameworks, may accommodate epigenome-edited varieties (USDA-APHIS, 2020; UK Parliament, 2023). The European Union’s deliberations over New Genomic Techniques regulation leave the status of dCas9 editing unsettled (European Commission, 2023; Voigt, 2023). Notably, dCas9 systems that leave no transgene in the final variety would likely face the lightest regulatory burden, reinforcing the engineering priority of editors that act transiently.

Three further factors will shape deployment. Biosafety assessment has no settled framework here, because the relevant hazards—off-target methylation, and pleiotropy through shared chromatin machinery—are invisible to the sequence-based screens used for nuclease editing and instead require methylome-scale characterization. Off-target epigenetic effects are a particular concern. dCas9-effector fusions can deposit marks at partially complementary sites, and such changes may be silent in controlled settings yet consequential under field stress. Public acceptance may prove more favorable than for transgenics because no foreign DNA persists and no sequence is altered, but this has not been tested empirically. Finally, the dCas9 and SunTag patent landscape is dense, and licensing cost rather than technical feasibility may determine whether these tools reach minor crops.

## 5 Conclusion

Epigenetic stress memory has the potential to become an agronomic asset. The biological framework is established: plants encode stress histories through DNA methylation, histone modifications, and small RNAs, and these can sometimes be transmitted to offspring. The dCas9 toolkit is maturing rapidly. The gap lies between the two: existing dCas9 fusions are not yet optimized for the distinctive RdDM-centric, type I/II memory logic of plants, and the durability, transgenerational stability, and fitness consequences of engineered marks remain underexplored. This review has catalogued what each effector class has and has not been shown to do in planta (Table 1) and identified the experiment that would most directly test whether Arabidopsis findings scale: dCas9-mediated H3K4me3 deposition at a type II memory promoter in rice, assessed for transgenerational stability, physiological cost, and field yield. Progress along these lines would move the field from a collection of proof-of-concept demonstrations toward a body of tools deployable in agricultural settings.
